# A Women-Centered Internet-Based Cognitive Behavioral Therapy for Depressive Symptoms (the 3D Program): Protocol for a Randomized Controlled Trial

**DOI:** 10.2196/99700

**Published:** 2026-08-19

**Authors:** Natalia Angarita-Osorio, Aitana García-Estela, Livia A Terraneo, Nathaly Nuñez Pazmiño, Noemí Haro, Oscar J Pozo, Francesc Colom

**Affiliations:** 1 Mental Health Research Group, Hospital del Mar Research Institute, Carrer Dr Aiguader, 88, Barcelona, Catalonia, 08003, Spain, 34 93 316 04 00 ext 1493; 2 Department of Psychiatry and Forensic Medicine, Faculty of Medicine, Universitat Autònoma de Barcelona, Avinguda de Can Domènech, Bellaterra, Catalonia, Spain; 3 Department of Medicine and Surgery, Centro de Investigación Biomédica en Red de Salud Mental, Madrid, Madrid, Spain; 4 Faculty of Medicine and Surgery, University of Insubria, Varese, Lombardy, Italy; 5 Applied Metabolomics Research Group, Hospital del Mar Research Institute, Barcelona, Catalonia, Spain

**Keywords:** depression, women’s health, gender equity, internet-based intervention, cognitive behavioral therapy, mental health teletherapy, biomarkers, randomized controlled trial, metabolomics

## Abstract

**Background:**

Depressive disorders are a leading cause of disability among women worldwide, yet existing interventions often fail to account for the biological and sociocultural factors shaping women’s risk and symptom presentation.

**Objective:**

In response, this randomized, single-blind, controlled trial will evaluate the efficacy of the 3D Program—a gender-sensitive, guided internet-based cognitive behavioral therapy (iCBT) intervention for women with moderate depressive symptoms.

**Methods:**

A total of 120 women presenting with moderate depressive symptoms (17-item Hamilton Depression Rating Scale score 17‐23), aged 18 to 45 years, will be randomized to receive either the 10-week 3D Program or informational video content, with both groups continuing treatment as usual. The 3D Program will integrate personalized therapist support, self-guided digital modules, and a moderated online group, with content cocreated alongside women with lived experience of depressive symptoms. The primary outcome will be the change in depressive symptom severity, measured by the 17-item Hamilton Depression Rating Scale at 12- and 26-week postbaseline assessments. Secondary outcomes will include functioning, quality of life, perceived stress, and menstrual-related distress. Additionally, the study will explore potential biological predictors of treatment response, analyzing metabolic markers related to tryptophan metabolism and adrenal steroid pathways across multiple biological matrices. Data analyses will use linear mixed-effects models and multivariate techniques to examine treatment effects and possible biomarker associations.

**Results:**

The trial was funded in October 2023 (grant PI23/00257, Spanish Ministry of Economy and Competitiveness), and ethical approval was obtained in February 2024. The trial was registered in July 2025 (ClinicalTrials.gov NCT07060690). Data collection began in March 2026 and is projected to conclude in August 2027. As of April 2026, a total of 8 participants have been enrolled. Data analysis has not yet commenced. The results are expected to be published in the summer of 2028.

**Conclusions:**

This trial aims to address critical gaps in accessible, personalized, and gender-sensitive mental health care. If effective, the 3D Program may serve as a scalable, cost-efficient adjunct to routine care for women with depressive symptoms in public health systems, while biomarker findings may lay the groundwork for future trials examining predictors of response.

## Introduction

Depressive disorders are consistently more prevalent among women than men worldwide [1], with depression ranking as the 6th and 13th leading cause of disability in women and men, respectively [2]. In Spain specifically, depression affects women at twice the rate of men [3]. Women also face sex- and gender-specific risk factors, including hormone-related depressive disorders—premenstrual dysphoric disorder, peripartum depression, and perimenopausal depression—and sociocultural stressors such as higher exposure to interpersonal stress, caregiving responsibilities, and gender-based discrimination [4-7].

Even when depression is detected, many individuals do not receive evidence-based treatments considered minimally adequate [8,9], due to limited access, structural deficiencies, social stigma, and cost barriers—challenges present not only in low- and middle-income countries but also in high-income settings such as Spain [10]. Among psychological treatments for depression, cognitive behavioral therapy (CBT) is the most widely studied globally and consistently recommended as a first-line psychological treatment in Spain [11,12]. Internet-based CBT (iCBT) has emerged as a scalable alternative, demonstrating comparable effectiveness to face-to-face therapy for mild-to-moderate depression when guided by human support [13]. However, digital interventions face significant challenges, such as low adherence and lack of personalization, which reduce both engagement and effectiveness [14,15].

This one-size-fits-all approach is especially problematic for women, whose depression is shaped by unique biological and sociocultural risk factors rarely addressed by current approaches [7,16]. Addressing these sex- and gender-related differences may require insights from emerging biological research [17]. Two metabolic pathways—tryptophan metabolism and adrenal steroidogenesis—show promise for understanding the biological contributors to depression and informing personalized treatment approaches. Inflammatory processes can shift tryptophan metabolism from serotonin production toward the kynurenine pathway, a mechanism linked to depressive symptoms [18,19]. Adrenal steroidogenesis, which regulates the stress response and produces corticosteroids and sex hormone precursors, shows dysregulation patterns implicated in sex differences in depression [20].

To address persistent gaps in accessible, personalized, and gender-sensitive mental health care, this study will conduct a randomized controlled trial (RCT) evaluating the efficacy of the 3D Program, a gender-sensitive, guided iCBT intervention for women with moderate depressive symptoms, which was cocreated with multidisciplinary experts and women with lived experiences. We hypothesize that participants receiving the 3D Program will experience greater reductions in depressive symptoms compared to controls. Additionally, we will explore metabolic biomarkers related to tryptophan and steroid pathways in relation to treatment response.

## Methods

### Study Design

**Figure 1.**
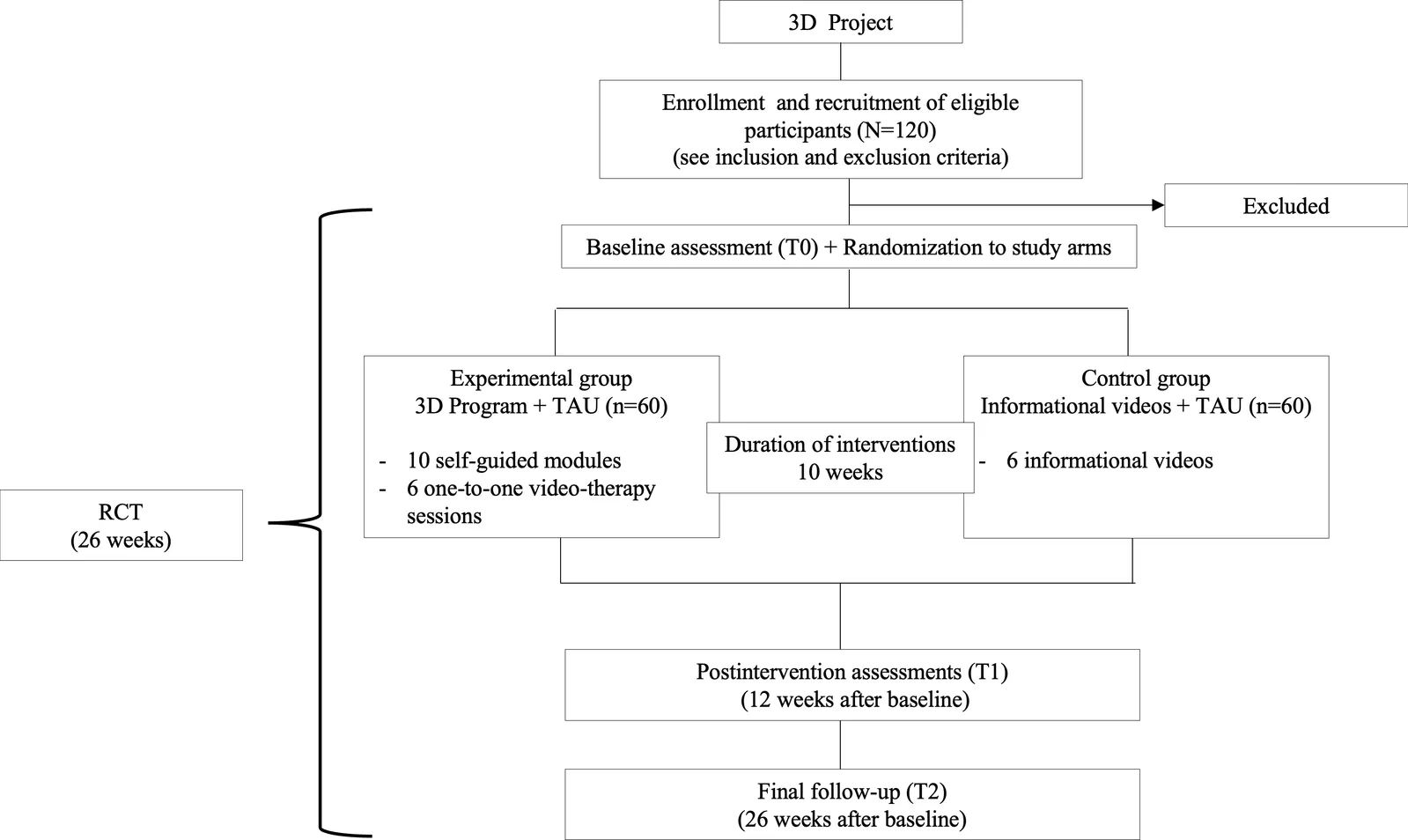
Flowchart of the study design*.* RCT: randomized controlled trial; TAU: treatment as usual.

This study is a randomized, single-blind, parallel-group, controlled trial testing the efficacy of the 3D Program—a gender-sensitive, guided iCBT intervention for women with moderate depressive symptoms delivered over 10 weeks compared to informational videos. The study outline is presented in Figure 1, and Checklist 1 contains the SPIRIT (Standard Protocol Items: Recommendations for Interventional Trials) checklist, as this protocol was developed in accordance with the SPIRIT guidelines [21].

The study encompasses 2 components: first, an evaluation of the intervention’s efficacy, and second, an exploratory investigation of metabolic signatures associated with treatment response, focusing on tryptophan and steroid-related pathways.

### Study Setting

#### Overview

Participants will be recruited from outpatient and community services within Hospital del Mar (Barcelona, Spain). The intervention will be delivered fully online: participants will engage with the program at home, while the therapists will conduct individual sessions from Hospital del Mar facilities to ensure privacy and compliance with ethical standards.

#### Patient and Public Involvement

The 3D Program was refined through a 2-phase cocreation and coadaptive process. Phase 1 involved a multidisciplinary panel of women (n=6; including a psychiatrist, a gynecologist, a medical doctor, and 3 psychologists) who developed the 10 self-guided modules. In phase 2, a total of 8 women with lived experience of depressive symptoms were recruited from existing research databases to participate in five 120-minute workshops. Using creative participatory dynamics, these sessions served to adapt the intervention content and test the beta platform, ensuring the clinical relevance, acceptability, and usability of the 3D Program.

### Participants

#### Recruitment

Recruitment will take place over a 12-month period and will conclude once the target sample size (N=120) is reached or the recruitment period ends, whichever occurs first. Recruitment strategies will include (1) attending regular clinical meetings; (2) displaying posters with a brief overview of the study in common areas of the hospital, as well as local libraries, civic centers, and university campuses within the Hospital del Mar catchment area; and (3) maintaining close contact with clinicians across inpatient, outpatient, and primary care settings to increase awareness of the study.

All participants meeting the inclusion criteria will be invited to participate by their treating clinical teams. If referred by nonmental health professionals, participants will be screened by a study psychologist or psychiatrist to confirm the presence and severity of depressive symptoms.

#### Eligibility

Eligible participants will be women (including both cisgender and transgender) aged 18 to 45 years (inclusive) who present with moderate depressive symptoms at baseline, defined as a score of 17 to 23 on the 17-item Hamilton Depression Rating Scale (HDRS-17). Additional inclusion criteria are fluency in Catalan or Spanish, regular internet access, and the ability to independently use a digital device (eg, smartphone, tablet, or computer).

Participants will be excluded if they are currently receiving psychological therapy, as concurrent structured psychotherapy would confound the estimation of the 3D Program’s effect; have participated in another interventional clinical trial for depression in the past 3 months; are pregnant or planning to become pregnant during the study period, given the distinctiveness of perinatal depression and because pregnancy-related hormonal fluctuations would confound the exploratory biomarker analyses; have cognitive impairment that would interfere with informed consent or study participation; or currently present with psychotic symptoms, active suicidal ideation, or substance use disorder. Women older than 45 years will be excluded to minimize potential confounding effects of perimenopausal hormonal changes. Participants with severe depression will be excluded due to safety considerations, as this population requires more intensive, closely monitored treatment than the 3D Program is designed to provide.

### Intervention

#### Experimental Group

Participants in the experimental group will receive the 3D Program—a gender-sensitive, guided iCBT intervention for women with depressive symptoms—delivered over 10 weeks alongside treatment as usual (TAU).

The 3D Program comprises 10 weekly self-guided modules, accessed via a secure website, incorporating video content, written materials, and interactive activities such as worksheets. Topics cover issues commonly experienced by women, including hormonal changes, caregiving, body image, and experiences of sexism. Prior to beginning the program, participants will receive an orientation video on how to navigate the platform.

In addition to the self-guided modules, participants will receive 6 individual online sessions with a trained female psychologist. The intervention adopts a personalized approach, allowing therapists to tailor each participant’s module sequence based on individual needs and clinical relevance. All therapists will complete training focused on the 3D Program and the principles of online psychological support.

Participants will also have access to an online peer support group, moderated by a trained female facilitator who will guide discussions to foster connection and safety. The group aims to reduce isolation, promote shared experiences, and challenge maladaptive beliefs such as “this only happens to me.”

The module content and group space were cocreated and coadapted through 5 workshop sessions with women with lived experience of depressive symptoms. Details on the content of the 3D Program modules are presented in Table S1 in Multimedia Appendix 1.

#### Control Group

Participants in the control group will receive 6 short informational video capsules based on selected content from the 3D Program modules in addition to TAU. Details on the content can be found in Table S2 in Multimedia Appendix 1.

To ensure an even distribution of content over the 10-week period, participants will be given access to 1 video every 14 days, facilitating regular engagement and structured exposure to the material. Control group participants will not receive interactive tasks or one-to-one sessions with a therapist.

All participants, regardless of group allocation, will continue their TAU under the supervision of their clinical care team. TAU comprises standard Spanish National Health System care, involving primary care coordination and specialist referrals to mental health services as needed. This encompasses psychoeducation, symptom monitoring, and first-line pharmacotherapy with selective serotonin reuptake inhibitors, plus short-term benzodiazepines if needed. Brief psychological interventions (eg, CBT) or more intensive psychotherapy may be provided for mild-moderate cases if indicated [12]. However, individuals currently receiving any such active treatment will be excluded from this trial to avoid potential confounding effects. No modifications to ongoing clinical care will be made for research purposes, ensuring that the 3D Program serves as a naturalistic adjunct to standard treatment.

#### Criteria for Discontinuing or Modifying Allocated Interventions

Participants may withdraw from the study at any point without providing a reason. The research team may also discontinue a participant’s involvement if continuation poses a risk to their well-being, or if significant protocol deviations occur (eg, inability to engage in the intervention as intended). Discontinuation may also occur in consultation with the treating clinical staff if significant clinical symptoms emerge that require alternative or intensified care, in which case participants will be referred to an appropriate service.

#### Strategies to Improve Adherence to Interventions

To promote engagement and adherence, participants in the experimental group will receive up to 6 one-to-one online video sessions with a trained female therapist, which introduces a human element that reinforces the therapeutic relationship and strengthens the therapeutic alliance. Additionally, time-sensitive notifications will be used to prompt participants when prescribed modules or worksheets remain incomplete. These automated reminders are designed to support program adherence, self-monitoring, and sustained participation throughout the intervention.

#### Relevant Concomitant Care Permitted or Prohibited During the Trial

Pharmacological treatment will be permitted, provided it was initiated at least 1 month before study entry. Concomitant pharmacotherapy (agent and dose) will be recorded at baseline, tracked for changes during subsequent assessments, and compared between the groups after randomization to detect any imbalances. Any concurrent psychological interventions or psychological care will not be permitted during the trial period to avoid confounding effects. Should any intensification of clinical care become necessary during the trial—including psychological interventions, changes to pharmacotherapy, specialist referrals, or crisis interventions—it will be documented in the trial log as part of the adverse event monitoring procedure. Where these intensifications reflect clinically significant symptoms worsening requiring alternate or intensive care, the participant may be discontinued from the trial in consultation with their treating team (see the *Criteria for Discontinuing or Modifying Allocated Interventions* section).

### Assignment of Interventions

#### Randomization

Participants will be randomized in a 1:1 ratio using the REDCap randomization module, which will automatically assign participants to either the experimental group or control group through simple randomization with equal allocation. The allocation sequence will be generated by an independent statistician or REDCap administrator who is not involved in participant recruitment, outcome assessment, or data analysis using the National Cancer Institute (NCI) Clinical Trial Randomization Tool [22]. This individual will be responsible for uploading the allocation table to REDCap, ensuring that allocation concealment is maintained throughout enrollment.

Following randomization, a designated unblinded team member (eg, intervention therapists) will access the group assignment in REDCap and will be responsible for providing participants with access credentials and instructions for the appropriate version of the intervention platform, according to their assigned group.

#### Blinding

This is a single-blind trial. Due to the nature of the intervention, participants will be aware of their group assignment after randomization. Likewise, therapists delivering the intervention will not be blinded, as is standard in psychological trials. To preserve blinding where possible and minimize bias, the following measures will be implemented: (1) outcome assessors will remain blinded to group allocation and will not have access to REDCap fields containing the randomization result; (2) data analysts will be blinded during analyses; and (3) all other stakeholders, including clinicians, statisticians, laboratory analysts, and the principal investigator, will not have access to group assignment information or the randomization sequence. Access control within REDCap will be configured using role-based permissions to ensure that only designated unblinded personnel can view or export the group allocation information.

### Data Collection

#### Overview

Data will be stored on a secure server at the Hospital del Mar Research Institute (HMRI), in compliance with EU and Spanish data protection regulations, namely the General Data Protection Regulation (GDPR) of May 25, 2018, and Organic Law 3/2018 of December 5, on the Protection of Personal Data and the Guarantee of Digital Rights.

#### Procedure

Assessments will be conducted at 3 time points: baseline (T0), postintervention (12 weeks after baseline, T1), and follow-up (26 weeks after baseline, T2).

The baseline assessment will include the collection of sociodemographic and clinical data, the administration of primary and secondary outcome measures, a gynecological consultation to assess hormonal and menstrual health, and the collection of biological samples.

At T1 and T2, follow-up assessments will include the administration of primary and secondary outcome measures as well as biological sample collection.

All psychological assessments will be carried out by a trained professional (psychologist, physician, psychiatrist, or psychiatric nurse), gynecological examinations will be performed by a licensed gynecologist (MD in obstetrics and gynecology), and biological samples will be collected by a registered nurse. Participant timeline, including the schedule of enrollment, interventions, and assessments, is outlined in Table 1. All professionals with direct participant contact—whether via teleconference or in-person—will be women. This decision was reached by consensus within the focus group of women with a lived experience of depression, who expressed that sensitive topics (eg, hormonal changes, gender discrimination, and sexuality in the context of depression) would be better understood by female professionals and that participants would feel more comfortable accordingly.

**Table 1.** SPIRIT (Standard Protocol Items: Recommendations for Interventional Trials) participant timeline.

Study procedure	Trial period					
	Preintervention			Intervention	Postintervention	Follow-up
Time point	–t0	T0	T0	—^a^	T1	T2
Weeks	0	1	2	3‐12	12	26
Enrollment						
Eligibility screen	*✓*					
Informed consent		✓				
Inclusion		✓				
Randomization		✓				
Interventions						
Intervention (3D Program)				✓		
Control group (informational videos)				✓		
Screening assessments						
Sociodemographic information		✓				
Medical and psychiatric history		✓				
Gynecological visit			✓			
Primary outcome						
HDRS-17^b^		✓			✓	✓
Secondary outcomes						
WHODAS 2.0^c^		✓			✓	✓
EQ-5D-5L^d^		✓			✓	✓
PSS^e^** **		✓			✓	✓
MEDI-Q^f^** **		✓			✓	✓
Biological samples						
Plasma			✓		✓	✓
Saliva			✓		✓	✓
Urine			✓		✓	✓
Nails			✓		✓	✓
Hair			✓		✓	✓

a Not applicable.

b HDRS-17: 17-item Hamilton Depression Rating Scale.

c WHODAS 2.0: World Health Organization Disability Assessment Schedule 2.0 (36-item version).

d EQ-5D-5L: EuroQol 5-Dimension 5-Level.

e PSS: Perceived Stress Scale.

f MEDI-Q: Menstrual Distress Questionnaire.

### Outcomes

#### Participant Characteristics and Clinical Data

Sociodemographic information will be self-reported, and clinical information will be collected by clinicians to describe the study population and adjust for potential confounding variables. Sociodemographic variables will include age, sex, gender identity, marital status, educational level, employment status, and household income.

Clinical data will comprise personal history of mental health diagnoses, current psychopharmacological treatment, and comorbid medical conditions. Adjustments to concomitant treatments will be recorded throughout the study. If changes occur, they will be evaluated and included in the analysis as covariates to adjust for potential confounding effects. Additional variables will include BMI derived from weight and height collected during the gynecological visit, as well as self-reported sleep, physical activity, and substance use.

Gynecological and reproductive health information will be gathered during a structured interview conducted as part of the gynecological consultation. This will cover menstrual, sexual, and reproductive history (eg, pregnancies, deliveries); diagnoses of gynecological conditions; and a physical examination. If any anomalies are detected, participants will be referred to their general practitioners or the appropriate specialist. Participants will not be excluded from the study if they do not express a willingness to quit.

#### Primary Outcome Measures

Change in depressive symptoms from baseline (T0) to 12 weeks (T1) and 26 weeks (T2) was assessed using the clinician-administered HDRS-17, which was validated in Spanish [23,24]. The HDRS-17 total score ranges from 0 to 52, with higher scores indicating more severe depression.

#### Secondary Outcome Measures

Secondary outcomes will include changes from baseline (T0) to 12 weeks (T1) and 26 weeks (T2) in overall functioning, health-related quality of life, perceived stress, and menstruation-related distress.

- Overall functioning: Assessed via the self-administered World Health Organization Disability Assessment Schedule 2.0 (WHODAS 2.0; 36-item version) [25,26]. It evaluates functioning across 6 domains, with higher scores indicating greater disability.
- Health-related quality of life: Measured by the self-reported EuroQol 5-Dimension 5-Level (EQ-5D-5L) [27,28], which assesses 5 dimensions of health on a 5-point scale. Responses are combined into a single index value ranging from below 0 (health states worse than death) to 1 (full health). A visual analogue scale is also included, ranging from 0 (worst imaginable health) to 100 (best imaginable health).
- Perceived stress: Evaluated using the self-reported Perceived Stress Scale (PSS) [29,30]. Items are rated on a 5-point Likert scale from 0 (never) to 4 (very often), yielding a total score from 0 to 56. Higher scores reflect greater perceived stress.
- Menstruation-related distress: Assessed via the 25-item self-reported Menstrual Distress Questionnaire (MEDI-Q) [31]. It evaluates symptoms such as pain, discomfort, psychological changes, gastrointestinal symptoms, and physiological alterations.

All instruments, except for the MEDI-Q, will be used in their Spanish validated versions. As the MEDI-Q is a recently developed tool not yet validated in Spanish, its use in this study provides an opportunity for initial cultural adaptation within this population.

### Biological Samples

#### Sample Types and Collection Procedures

Biological analysis will be conducted exclusively on specimens collected from cisgender women participants. Biological specimens to be collected include plasma, urine, saliva, fingernails, and hair. All collection procedures will adhere to standard operating procedures, with participant safety and comfort as priorities. Specific procedures for each biological sample are as follows:

- Plasma: Blood samples will be collected using EDTA K2 plastic tubes (13 × 75 mm, 4 mL). Two tubes (totaling 8 mL) will be collected per participant on the day of the assessment visit.
- Urine: Participants will self-collect approximately 50 mL of midstream urine the night before their scheduled assessment visit, using a sterile 60-mL polypropylene container. The sample will be refrigerated overnight at the participant’s home and transported to the research facility on the day of the visit.
- Saliva: Participants will self-collect saliva samples the night before the assessment visit using 2 Salivette Cortisol collection devices. Samples will be refrigerated overnight at the participant’s home and transported to the research facility on the day of the visit.
- Fingernails: Participants will clip the distal ends of their fingernails at home before the assessment visit. Samples will be collected in sealed paper envelopes.
- Hair: A strand of hair (approximately 0.5 cm in diameter, or 50 mg for short hair) will be cut from the posterior vertex as close to the scalp as possible by trained personnel. The root end will be marked with a hair tie. Samples will be collected in sealed paper envelopes.

#### Sample Transport and Biobanking

All samples will be collected or delivered to the Hospital del Mar facilities and subsequently processed, analyzed, and stored at the Parc de Recerca Biomèdica de Barcelona (PRBB) facilities and the Cryopreservation Service at the HMRI. Plasma, urine, and saliva will be initially stored at −20 °C and transferred to −80 °C for long-term storage. Hair and nail samples will be stored at room temperature in a locked cabinet under humidity-controlled conditions until processing.

#### Sample Processing Protocols

The collected biological samples will be processed according to standardized protocols prior to laboratory analysis, as outlined below:

- Plasma, urine, and saliva: All samples will be centrifuged upon arrival at the laboratory. Aliquots will be prepared as necessary. Chemical treatment will be applied if specified in the manufacturer’s protocol for the collection device (eg, Salivette for saliva). All reprocessing procedures will follow the manufacturer’s instructions.
- Fingernail samples will be washed with an organic solvent to remove external contaminants, then dried and mechanically ground using a ball mill to produce a homogeneous powder suitable for downstream analysis.
- Hair samples will undergo solvent-based washing to remove surface contamination, followed by drying and mechanical pulverization using a ball mill to prepare for analytical procedures.

#### Laboratory Oversight and Quality Control

All biochemical analyses will be performed internally in collaboration with the Applied Metabolomics Research Group (LIMA) at the HMRI. Quality control (QC) procedures will include pooled QC samples from healthy donors. At least 3 QC aliquots will be analyzed with each analytical batch.

#### Analytical Methods

The quantification of targeted metabolites in the tryptophan and steroid hormone pathways will be conducted using liquid chromatography-tandem mass spectrometry (LC-MS/MS) across all available biological matrices. This includes the measurement of tryptophan, kynurenine, serotonin, kynurenic acid, and relevant metabolic ratios (eg, kynurenine or tryptophan), as well as estradiol, testosterone, progesterone, cortisol, and their derivatives (eg, 20α-DHE, 20β-DHE). Data acquisition and processing will be performed using MassLynx 4.2 (version 4.2; Waters Corp.) and the TargetLynx application manager (Waters Corp.). To minimize interbatch variability, all samples will be analyzed in a single run once collection is complete. Given the sample size, biomarker analyses will be considered exploratory and hypothesis-generating, and the study is not powered to detect definitive associations.

#### Adverse Events

Participant safety will be monitored throughout the trial. Therapists will assess symptom worsening and psychological distress during individual sessions, with all events recorded in the trial log, and evaluated by the principal investigator or a designated clinician for seriousness, expectedness, and relatedness to the intervention. Per International Council for Harmonisation (ICH) guidelines [32], serious adverse events (SAEs) are defined as any untoward medical occurrence resulting in death, life-threatening outcomes, hospitalization, significant disability, or other medically important conditions.

In this trial, psychological SAEs include suicide attempts, imminent suicidal or homicidal intent, self-harm requiring medical treatment, psychiatric hospitalization, or severe mental state deterioration causing significant impairment. These criteria apply to all events occurring during or after participation, regardless of causality.

SAEs will be reported to the principal investigator within 24 hours and to the research ethics committee as required. Significant safety concerns will trigger a clinical review to determine the need for safety plans, urgent referral, or trial discontinuation. Imminent suicide risk will immediately activate local suicide-risk protocols, including same-day clinical assessment and emergency or crisis service referral.

### Data Management

Data will be collected, and managed using the REDCap electronic data capture system [33], hosted by the HMRI, which provides a secure and reliable platform for managing research data. Access to the data will be safeguarded by a password-protected system, with user permissions defined by individual roles and access levels assigned through unique user IDs.

Original signed consent forms will be securely stored in numerical order in locked cabinets at the study site. These documents will be retained for 5 years following the conclusion of the study, with access restricted to authorized personnel only.

### Data Analysis

#### Sample Size Calculation

A total sample size of 120 participants (60 per group) is required. This estimation was calculated based on a design with 2 groups and 3 measurement points. Assuming an effect size of *f* of 0.15, a significance level of *α* of .05, and a power of 0.90, with a correlation among repeated measures of 0.5 and assumed sphericity (*ε*=1), the minimum sample size estimated was at 96 patients. This number was increased to 120 to account for an anticipated 20% attrition rate. The sample size was determined using G*Power version 3.1.7.

#### Statistical Analysis

The statistical analysis plan was developed in collaboration with 2 biostatisticians. The analysis will be performed using SPSS Statistics (version 26; IBM Corp.).

Descriptive statistics will summarize sociodemographic and biomedical variables at baseline. Metabolomic variables will be analyzed across all time points; a complete list of the metabolomic variables analyzed in each matrix is provided in Table S3 in Multimedia Appendix 1. Continuous variables will be presented as means and SDs or medians and IQRs, depending on the distribution, while categorical variables will be reported as frequencies and percentages.

#### Outcome Analysis and Longitudinal Modeling

Primary and secondary outcomes will be analyzed using an intention-to-treat (ITT) approach, with all participants analyzed according to their original randomization group. Linear mixed-effects models (LMMs) will be used to examine changes over time and between groups for the HDRS-17, WHODAS 2.0, EQ-5D-5L, PSS, and MEDI-Q scores. These models will include fixed effects for group, time, and their interaction, with a random intercept for participants to account for within-subject correlation over time.

While change scores may be calculated for descriptive or interpretative purposes, the LMMs will serve as the primary inferential approach. In the event of a significant group × time interaction, post hoc simple effect analyses will be conducted to evaluate between-group differences at a specific time point and within-group changes from baseline, using estimated marginal means with appropriate corrections for multiple comparisons.

#### Predictors and Covariate Analysis

Additional analyses will identify baseline predictors of clinical improvement using LMMs and multiple linear regression. To address the dynamic nature of psychological factors, perceived stress (PSS) and menstrual distress (MEDI-Q) will be modeled as time-varying covariates within the LMM framework rather than as static baseline moderators. This allows for the assessment of whether longitudinal fluctuations in these variables are associated with concurrent changes in depressive symptoms. Furthermore, exploratory multilevel mediation analyses may be conducted to examine if changes in PSS or MEDI-Q partially mediate the intervention’s effect on HDRS-17 trajectories.

#### Metabolomics

Partial least squares regression and multiple regression analyses will be used to model associations between metabolite profiles, their changes over time, and changes in depressive symptoms. Variable importance in projection scores and cross-validation techniques will be used to evaluate model performance and identify key predictive biomarkers. Additionally, metabolomic changes will be assessed through exploratory factor analysis, grouping variables into latent factors. It should be noted that the study was originally conceptualized and powered as a confirmatory superiority RCT; therefore, the metabolomics substudy should be considered secondary and may be limited by this design. Nevertheless, given the scarcity of data in this biological field of research, we believe it is worthwhile to conduct this post hoc exploratory proof-of-concept study.

#### Missing Data

Following the ITT principle, LMMs will be applied without imputation, as they appropriately handle missing data under the missing at random assumption, and imputation may introduce unnecessary bias. Cluster and factor analyses will also be conducted without imputation, as they rely solely on baseline data, assuming that missingness is minimal and not systematic. Multiple imputation will be performed only for analyses that require it, such as multiple regression models and partial least squares regression.

All analyses will be 2-tailed, with statistical significance set at *P*<.05.

### Ethical Considerations

#### Approval and Dissemination

This study will be conducted in accordance with the Declaration of Helsinki 1964 and its following amendments (75th WMA General Assembly, Helsinki, Finland, October 2024). Ethical approval was obtained from the Drug Research Ethical Committee of the HMRI (2023/11236/I). The study will adhere to the CONSORT (Consolidated Standards of Reporting Trials) guidelines [34] for the reporting and publication of results. Once the trial is completed, results will be published in international peer-reviewed journals.

#### Informed Consent

All participants will receive detailed written and oral information about the study (ie, objectives, procedures, risks, and benefits) before enrollment. Once participants are informed, they will be asked to provide written informed consent for study inclusion. Specific consent will be obtained for the collection and future use of the biological samples; participants will be given the opportunity to decide which biological sample they wish to provide (ie, they may consent to provide some but not all biological samples).

#### Protocol Amendments

Any substantial modifications to the protocol, such as changes to eligibility criteria, outcome measures, or the analysis plan, will be promptly communicated to all participants, the relevant ethics committee, the trial registry, and all investigators involved. All amendments will be documented in detail, dated, and maintained as part of the official study record.

#### Confidentiality

Participant confidentiality and data security will be safeguarded in accordance with Spanish data protection legislation, including Organic Law 3/2018, of December 5, on the Protection of Personal Data and the Guarantee of Digital Rights. Each participant will be assigned a unique identification number, which will be used throughout all study phases. The mapping between the identification number and personal identifiers will be stored separately in an encrypted, password-protected file kept offline at the HMRI. Original paper records will be stored in locked cabinets on-site.

Participants will be identified only by their identification number and a secondary code or username, which will also serve as their login credentials for accessing the intervention platform. Upon login, participants will only provide their first names to personalize the experience; no additional personally identifying information will be collected via the platform. All data transmitted through the platform will be handled using appropriate technical and organizational safeguards.

The study database will be hosted on a secure physical server located at HMRI facilities. All data will be pseudoanonymized and used exclusively for study-related analyses. This pseudoanonymization will be maintained throughout the trial and in all subsequent data handling or publication.

#### Participant Safety

All data collection procedures will be carried out by trained research personnel with appropriate qualifications and certifications for administering the study assessments. A registered nurse will perform venipuncture and collect biological samples. All sample collections will adhere to established standard operating procedures approved by the HMRI Drug Research Ethical Committee.

Any unexpected clinical findings identified during the gynecological examination or through biological analyses will be addressed appropriately. If any such result requires immediate medical attention, the participant will be informed, and the relevant findings will be referred to their treating physician or general practitioner for further clinical evaluation and care.

#### Coordination and Oversight

A committee comprising senior investigators (FC and OJP) will oversee study progress. No independent data monitoring committee will be established, as the intervention is considered to be of minimal risk. The research team will hold monthly meetings to review safety and adherence to the protocol.

No interim analyses are planned. The trial will continue until full recruitment is achieved, unless safety concerns warrant early termination, although such events are anticipated to be rare given the low-risk nature of the intervention. The research team will conduct regular safety reviews to promptly identify and manage any potential risks.

## Results

The trial was funded in October 2023 (grant PI23/00257, Spanish Ministry of Economy and Competitiveness), and ethical approval was obtained in February 2024 (2023/11236/I, HMRI Drug Research Ethical Committee). The trial was registered in July 2025 (ClinicalTrials.gov NCT07060690). Before data collection began, the intervention modules were finalized through a cocreation and coadaptive process involving women with lived experience of depressive symptoms. The delivery platform was developed by a multidisciplinary team of 5 members (1 software engineer and 4 psychologists) and refined following a pilot phase with women with lived experience of depressive symptoms (n=8) to address technical, accessibility, and usability concerns. Data collection began in March 2026 and is projected to conclude in August 2027. As of April 2026, a total of 8 participants have been enrolled. Data analysis has not yet commenced. The results are expected to be published in the summer of 2028.

## Discussion

This RCT will evaluate the efficacy of the 3D Program, a gender-sensitive, guided iCBT intervention for women with moderate depressive symptoms. In addition to assessing clinical symptom reduction, the study also aims to investigate the potential biological mechanisms of treatment response through metabolomic and hormonal analyses. To our knowledge, this is among the first online psychological interventions specifically designed for women with depressive symptoms outside the perinatal period. Given that depressive symptoms disproportionately affect women and are shaped by both biological and sociocultural factors, the 3D Program addresses a longstanding gap in accessible, inclusive, and gender-sensitive mental health care. The intervention content was cocreated with women with lived experience of depressive symptoms—an approach that enhances ecological validity and acceptability of the program. This participatory approach may also improve uptake and real-world sustainability if efficacy is demonstrated.

A novel feature of this trial is the seminal search for the role of sex hormones and glucocorticoids in moderating treatment effects. The integration of metabolic profiling, focusing on tryptophan and steroid pathways, may offer preliminary insights into biological factors associated with individual differences in treatment response. This exploratory approach aligns with emerging models of personalized psychiatry and may contribute to the identification of biomarkers associated with therapeutic efficacy in depression treatment.

If effective, the 3D Program could serve as a cost-efficient and scalable adjunct to routine care within the public Spanish National Health System, with potential for adaptation and implementation in other publicly funded health care systems. It could reduce clinician burden and broaden access to evidence-based care, while empowering users through enhanced self-management. Its practical implementation—with therapist training deliverable in a single workshop—supports feasibility for integration into standard care pathways. Moreover, the modular and adaptable design of the program may facilitate future use in other populations, including older women and gender-diverse individuals. It may also allow integration with complementary care modalities such as pharmacotherapy or face-to-face psychological interventions, thereby broadening its applicability and impact.

Despite these strengths, some limitations should be considered. First, while the intervention is multicomponent and tailored, it does not isolate which elements are most effective. Second, participant motivation may introduce selection bias, as participants may be more engaged or health-literate than the general population, potentially limiting the generalizability of the findings. While this may enhance adherence—mitigating the high dropout rates often associated with digital interventions—the study’s use of ITT analysis and a conservative attrition estimate in the sample size calculation aims to mitigate this concern. Third, biological sampling introduces challenges related to adherence and timing; some samples must be self-collected at home, relying on participant compliance. More rigorous circadian control requiring multiple visits was deemed infeasible and potentially detrimental to recruitment and retention. Fourth, while the eligibility criteria optimize participant safety and data clarity, they limit the generalizability of the findings. These criteria were selected to align the intervention with an appropriate symptom severity level and minimize confounding variables in the biomarker analyses. Consequently, further research will be required to determine if these results extend to other age groups, perinatal or perimenopausal depressed populations. Finally, the effectiveness of digital delivery may be affected by platform usability; to address this, the platform underwent usability testing during the cocreation phase, and technical support and a dedicated helpline will be available to all participants.

In conclusion, this study evaluates a novel, scalable, and biologically informed approach to digital mental health care tailored for women. If successful, it may help bridge persistent gender gaps in access and treatment outcomes and inform the future development of personalized, inclusive interventions in psychiatry.

## Supplementary material

10.2196/99700 Multimedia Appendix 1 Overview of the 3D Program, informational videos, and metabolomic variables.

10.2196/99700 Checklist 1 SPIRIT checklist

10.2196/99700 Peer Review Report 1 Grant proposal final review from the funding institution Instituto de Salud Carlos III.

10.2196/99700 Peer Review Report 2 Grant proposal scientific review from the funding institution Instituto de Salud Carlos III.

10.2196/99700 Peer Review Report 3 Grant proposal strategic review from the funding institution Instituto de Salud Carlos III.

## Abbreviations:

CBT: cognitive behavioral therapy
CONSORT: Consolidated Standards of Reporting Trials
EQ-5D-5L: EuroQol 5-Dimension 5-Level
GDPR: General Data Protection Regulation
HDRS-17: 17-item Hamilton Depression Rating Scale
HMRI: Hospital del Mar Research Institute
iCBT: internet-based cognitive behavioral therapy
ICH: International Council for Harmonisation
ITT: intention-to-treat
LC-MS/MS: liquid chromatography-tandem mass spectrometry
LIMA: Applied Metabolomics Research Group
LMM: linear mixed-effects model
MEDI-Q: Menstrual Distress Questionnaire
NCI: National Cancer Institute
PRBB: Parc de Recerca Biomèdica de Barcelona
PSS: Perceived Stress Scale
QC: quality control
RCT: randomized controlled trial
SAE: serious adverse event
SPIRIT: Standard Protocol Items: Recommendations for Interventional Trials
TAU: treatment as usual
WHODAS 2.0: World Health Organization Disability Assessment Schedule 2.0
