# Supplementary material for: A Women-Centered Internet-Based Cognitive Behavioral Therapy for Depressive Symptoms (the 3D Program): Protocol for a Randomized Controlled Trial
Source: JMIR Res Protoc. 2026 Aug 19;15:e99700. doi: 10.2196/99700 (PMC13489283; doi:10.2196/99700)
Supplement: Supplementary file 1 [file resprot-v15-e99700-s001.pdf]

Supplementary material

Table S1. Content of the 3D Program

Week	Content of the 3D Program.
**1**	**M1. Thinking, feeling, and doing**. Understanding the connection between thoughts, emotions, and behaviors.<br>**WS1**. Self-monitoring of daily activities and mood fluctuations to identify patterns.
**2**	**M2. Sleep and depression.** Exploring the impact of sleep habits on mood and depressive symptoms.<br>**WS2**. Daily challenges to incorporate small, actionable steps to improve sleep habits.
**3**	**M3. Planning and doing enjoyable things**. Understanding the importance of planning and (re)integrating pleasurable activities into daily routines.<br>**WS3**. Balancing responsibilities and leisure activities within daily schedules.
**4**	**M4. Getting things done.** Introducing problem-solving strategies to manage tasks without feeling overwhelmed.<br>**WS4**. Identifying to-do tasks and planning solutions to achieve goals.
**5**	**M5. Identifying negative thoughts.** Understanding the ABC model (Antecedents, Beliefs and Consequences) and how negative thoughts influence mood.<br>**WS5**. Applying the "ABC" model to analyze personal experiences.
**6**	**M6. Changing negative thoughts.** Learning to question negative thoughts and replacing them with realistic alternatives.<br>**WS6**. Creating alternative thoughts based on the analysis from M5.
**7**	**M7. Healthy lifestyle.** Exploring how physical health and healthy habits influence mood and behavior.<br>**WS7**. Daily challenges to incorporate small steps every day to improve your habits (i.e., diet, exercise).
**8**	**M8. Hormonal cycle and depression.** Understanding the relationship between the menstrual cycle and mood alterations, sexual function and fertility issues.<br>**WS8**. To be developed in the focus group.
**9**	**M9**. **Coping Strategies for Overcoming Adversity.** Developing problem-solving and emotional coping skills to address common struggles (e.g., mental load, caregiving responsibilities, sexism...).<br>**WS9**. Identifying coping skills and useful strategies. To be developed in the focus group.
**10**	**M10. Depression triggers.** Identifying personal warning signs linked to the emergence of depression and how to address them.<br>**WS10**. Identifying coping skills and problem-solving strategies.

Note. M: module; WS: worksheets

Table S2. Content of the informational videos for the control group

Week	Content of the informational videos
**1**	**M1. Thinking, feeling, and doing**. Understanding the connection between thoughts, emotions, and behaviors.
**3**	**M2. Sleep and depression.** Exploring the impact of sleep habits on mood and depressive symptoms.
**5**	**M3. Planning and doing enjoyable things**. Understanding the importance of planning and (re)integrating pleasurable activities into daily routines.
**7**	**M5. Identifying negative thoughts.** Understanding the ABC model (Antecedents, Beliefs and Consequences) and how negative thoughts influence mood.
**9**	**M7. Healthy lifestyle.** Exploring how physical health and healthy habits influence mood and behavior.
**10**	**M8. Hormonal cycle and depression.** Understanding the relationship between the menstrual cycle and mood alterations, sexual function and fertility issues.

Note. M: module

Table S3. Metabolomic variables analyzed in each matrix across all time points (T0, T1, and T2).

**Metabolite**	**Plasma**	**Urine**	**Saliva**	**Hair**	**Nails**
Cortisol (F)	✓	✓	✓	✓	✓
20α-DHF	✓	✓	✓	✓	
20β-DHF	✓	✓	✓	✓	
Cortisone (E)	✓	✓	✓	✓	✓
20α-DHE	✓	✓	✓	✓	✓
20β-DHE	✓	✓	✓	✓	✓
5α-THF		✓			
5β-THF		✓			
α,α-Cortol		✓			
α,β-Cortol		✓			
β,α-Cortol		✓			
β,β-Cortol		✓			
6OH-F		✓			
Corticosterone	✓	✓	✓		
11-dehydrocorticosterone	✓	✓	✓		
DHEAS	✓		✓		
Pregnenolone-S	✓		✓		
Testosterone	✓	✓	✓	✓	✓
Androstenedione	✓	✓	✓	✓	✓
Androsterone		✓			
Etiocholanolone		✓			
Progesterone	✓		✓		
17HP		✓			
Pregnantriol		✓			
Estradiol	✓		✓		
Tryptophan	✓	✓	✓		✓
Kynurenine	✓	✓	✓		✓
Kynurenic acid	✓	✓	✓		
3OH-Kynurenine	✓	✓			
Serotonin (5HT)	✓	✓	✓		✓
Melatonin			✓		✓
**Metabolic ratios**	**Plasma**	**Urine**	**Saliva**	**Hair**	**Nails**
F/E	✓	✓	✓	✓	✓
20α-DHF/F	✓	✓	✓	✓	
20β-DHF/F	✓	✓	✓	✓	
20α-DHE/E	✓	✓	✓	✓	✓
20β-DHE/E	✓	✓	✓	✓	✓
F metabolism	✓	✓	✓	✓	✓
6OH-F/F		✓			
Progesterone/Estradiol	✓		✓		
Kyn/Trp	✓	✓	✓		✓
5HT/Trp	✓	✓	✓		✓
Kyn/5HT	✓	✓	✓		✓
3OHKyn/Kyn	✓	✓			
KA/Kyn	✓	✓	✓		
