# Supplementary material for: A Women-Centered Internet-Based Cognitive Behavioral Therapy for Depressive Symptoms (the 3D Program): Protocol for a Randomized Controlled Trial
Source: JMIR Res Protoc. 2026 Aug 19;15:e99700. doi: 10.2196/99700 (PMC13489283; doi:10.2196/99700)
Supplement: Supplementary file 3 [file resprot-v15-e99700-s003.pdf]

DATOS GENERALES

**EXPEDIENTE**	PI23/00257
**INVESTIGADOR PRINCIPAL**	COLOM VICTORIANO, FRANCESC
**COINVESTIGADOR PRINCIPAL**	,
**TÍTULO**	Las mujeres luchan contra la depresión (WFD): una intervención basada en un programa online con interacción humana. Estudio de su eficacia e implicación metabolómica en la respuesta personalizada.
**CENTRO DE REALIZACIÓN**	INSTITUTO MAIMONIDES DE INVESTIGACION BIOMEDICA DE CORDOBA (IMIBIC)
**CENTRO SOLICITANTE**	FUNDACION INSTITUTO HOSPITAL DEL MAR DE INVESTIGACIONES MEDICAS (FIMIM)
**COMISIÓN TÉCNICA DE EVALUACIÓN**	Enfermedades neurológicas y mentales

INFORME DE EVALUACIÓN (máx.100 puntos)

PUNTUACIONES

VALORACIÓN EQUIPO	VALORACIÓN PROYECTO	DISCAPACIDAD	EMERGENTE	TOTAL
22,00	58,00	0,00	0,00	80,00

VALORACION DEL EQUIPO DE INVESTIGACIÓN (máx. 30 puntos).

• Institución del IP/COIP (IIS / OPI /centro (AP o hospital) SNS en provincia sin IIS)(S=2 puntos: N=0)	S
• IP/COIP contratado Miguel Servet/contratado Juan Rodés/ investigador clínico asistencial. (S=2; N=0)	N
• Edad IP/COIP menor /igual a 45 años. (S=2; N=0)	N
• Equilibrio de género en la composición del equipo investigador. (S=2; N=0)	S
• Se acredita participación ciudadana en el proyecto (S=1; N=0)	S
• IP/COIP con capacidad demostrada de atracción de talento (S=1; N=0)	S
• Perfil del IP /COIP: (De 0-12 puntos)	11,00
• Equipo investigador: (De 0 a 5 puntos)	3,00
• Capacidad formativa del IP/COIP del equipo: (De 0 a 3 puntos)	2,00

PUNTUACIÓN TOTAL EQUIPO 22,00

VALORACIÓN DEL PROYECTO (máx. 70 puntos).

- **Relevancia: (De 0 a 15 puntos)**: contribución esperada al avance de la ciencia en salud; estimación formal/informal del impacto (en salud, económico y social) por la implementación de sus resultados. — 15,00

- **Aplicabilidad: (De 0 a 15 puntos):** aplicabilidad directa o diferida, justificar la aplicabilidad directa, en caso de aplicabilidad diferida, considerar si se identifican retos regulatorios/organizativos a su implementación; vulnerabilidad del problema abordado; requiere modificaciones regulatorias u organizativas mayores. — 13,00

- **Capacidad de transferencia del proyecto: (De 0 a 8 puntos)**: actuaciones previstas orientadas a la transferencia a la práctica clínica de los resultados, y su potencial incorporación en cartera de servicios; transferencia al sector productivo; limitaciones mayores para la transferencia efectiva. — 6,00

- **Perspectiva de género: (De 0 a 2 puntos):** Tener en cuenta para la valoración de este ítem si está incorporada la perspectiva de género en el diseño del proyecto y si es adecuado el planteamiento. — 2,00

- **Calidad científica: (De 0 a 7 puntos):** novedad de la propuesta, originalidad en el abordaje conceptual y/o experimental de la misma, pertinencia; efecto incentivador, análisis actualizado del estado de conocimiento; integración en una línea de investigación estable con proyectos previos que se detallan; capacidad de transferencia directa de los resultados esperados. — 5,00

- **Calidad metodológica (De 0 a 8 puntos):** alineamiento entre hipótesis y objetivos, formulados de forma clara y concisa; adecuación metodológica del diseño, variables y plan de análisis estadístico a los objetivos propuestos; tamaño muestral, justificación formal/informal; identificación de fuentes de sesgos y previsión para su control; propuesta de plan de gestión de datos (PGD). — 5,00

- **Viabilidad: (De 0 a 15 puntos):** adecuación del equipo al proyecto, adecuación entre sus competencias y las actividades experimentales a desarrollar, experiencia previa; plan de trabajo, distribución de tareas y cronograma; identificación de riesgos y planificación de su control; infraestructuras disponibles, pertinencia y capacidad para su gestión; adecuación del presupuesto solicitado a los objetivos del proyecto; experiencia acreditada en gestión de proyectos, valoración específica en propuestas coordinadas o con presupuesto solicitado>300.000 euros. — 12,00

PUNTUACIÓN TOTAL PROYECTO — 58,00

**IP / COIP CON GRADO DE DISCAPACIDAD ≥ 33%**	NO
**IP/COIP EMERGENTE Y CUMPLE LOS CRITERIOS DE PRIORIDAD**	NO
**¿SE INSTA AL BENEFICIARIO PARA QUE REFORMULE SU SOLICITUD EN PERIODO DE ALEGACIONES PARA AJUSTAR SUS COMPROMISOS Y CONDICIONES A LA SUBVENCIÓN OTORGABLE?**	NO
**¿ESTE PROYECTO ES SUSCEPTIBLE DE EVALUACIÓN CIENTÍFICA A LOS NUEVE MESES DEL INICIO DEL PERIODO DE EJECUCIÓN?**	NO

VALORACIÓN

Valoración total

Una vez analizados y discutidos los distintos informes previos en la comisión técnica evaluadora y armonizados los distintos aspectos científicos y estratégicos del proyecto, se concluye la siguiente valoración:
Proyecto relevante en la temática que afronta, muy necesaria desde el punto de vista asistencial, transferible. Se detectan algunas deficiencias metodológicas que convendría abordar; se detectan algunos riesgos en relación con la participación (por "brecha digital", o el sostenimiento financiero de una intervención online, que no se discuten. El presupuesto solicitado para objetivos secundarios es desproporcionado, por el peso de estos objetivos en el proyecto.

PUNTUACIÓN TOTAL 80,00

Los datos personales que aparecen en este documento sólo serán utilizados para los fines previstos en la correspondiente Convocatoria o para su tratamiento automatizado con fines estadísticos con las garantías previstas en Reglamento (UE) 2016/679 del Parlamento Europeo y del Consejo de 27, de abril de 2016, relativo a la protección de las personas físicas en lo que respecta al tratamiento de datos personales y a la libre circulación de estos datos y por el que se deroga la Directiva 95/46/CE (Reglamento general de protección de datos) y en la Ley Orgánica 3/2018, de 5 de diciembre, de Protección de Datos Personales y garantía de los derechos digitales.
