# Supplementary material for: A Women-Centered Internet-Based Cognitive Behavioral Therapy for Depressive Symptoms (the 3D Program): Protocol for a Randomized Controlled Trial
Source: JMIR Res Protoc. 2026 Aug 19;15:e99700. doi: 10.2196/99700 (PMC13489283; doi:10.2196/99700)
Supplement: Supplementary file 4 [file resprot-v15-e99700-s004.pdf]

DATOS GENERALES

**EXPEDIENTE**	PI23/00257
**INVESTIGADOR PRINCIPAL**	COLOM VICTORIANO, FRANCESC
**COINVESTIGADOR PRINCIPAL**	,
**TÍTULO**	Las mujeres luchan contra la depresión (WFD): una intervención basada en un programa online con interacción humana. Estudio de su eficacia e implicación metabolómica en la respuesta personalizada.
**CENTRO DE REALIZACIÓN**	INSTITUTO HOSPITAL DEL MAR DE INVESTIGACIONES MEDICAS (IMIM)
**CENTRO SOLICITANTE**	FUNDACION INSTITUTO HOSPITAL DEL MAR DE INVESTIGACIONES MEDICAS (FIMIM)
**COMISIÓN TECNICA DE EVALUACIÓN**	Enfermedades neurológicas y mentales

INFORME SÍNTESIS CIENTIFICO-TÉCNICO (máx. 50 puntos)

A) VALORACIÓN DEL EQUIPO DE INVESTIGACIÓN:(máx. 20 puntos en este apartado):

1º Perfil del IP /COIP (De 0-12 puntos): dar hasta 8 puntos para proyectos que cumplan con los criterios y subir a 12 sólo en casos de recorridos excelentes de los IP/COIP.

2º Equipo investigador: (De 0 a 5 puntos): adecuación entre competencias/capacidades en el ámbito de conocimiento del proyecto y las actividades previstas para su desarrollo.

3º Capacidad formativa del IP/COIP y del equipo: (De 0 a 3 puntos): dirección de Tesis Doctorales, dirección de Máster; captación de financiación de RR. HH. en convocatorias competitivas, si tiene residentes o rotantes a su cargo.

Justifique brevemente la valoración otorgada en cada uno de los tres apartados, respecto a la **valoración científica del equipo**

El investigador principal tiene un recorrido de reconocido prestigio, tiene capacidad de liderazgo y de gestión de proyectos, y le avala una trayectoria de publicaciones al más alto nivel. Pertenece a estructuras estables y tiene un buen grado de internacionalización. Hay proyectos previos relevantes e integración en estructuras estables de investigación.
El resto del equipo es de un perfil más clínico con experiencia variable en investigación (distintas generaciones de investigadores), aunque podría haberse incluido algún investigador con más trayectoria y proyectos en la parte biológica (metabolómica), que solo está cubierta por un técnico de laboratorio. Un inconveniente es la parte de formación, con menos evidencias que en el resto de apartados y que no concuerda con la experiencia investigadora del IP.

B) VALORACIÓN DEL PROYECTO: (máx. 30 puntos en este apartado):

1º Calidad científica: (De 0 a 7 puntos): novedad de la propuesta, originalidad en el abordaje conceptual y/o experimental de la misma, pertinencia; efecto incentivador, análisis actualizado del estado de conocimiento; integración en una línea de investigación estable con proyectos previos que se detallan; capacidad de transferencia directa de los resultados esperados.

Justifique la valoración otorgada

El proyecto no es completamente novedoso, pues parte de la ya conocida herramienta online iFitgthDepression, pero sí original al incluir nuevos módulos para controlar ciclos menstruales / detección y gestión de micromachismos. Además se realizarán analíticas a nivel basal para la evaluación de parámetros hormonales y metabólicos relacionados para predecir la respuesta a la herramienta (aunque la justificación para esta parte "biológica" no está muy desarrollada).
Se justifica a partir de una necesidad real de facilitar la intervención, creando un sistema más accesible. Es previsible que obtengan resultados favorables. Se define con claridad la eficacia de la intervención pero ésta presenta un elevado coste económico y con elevados costes de mantenimiento que podrían dificultar su continuidad. Uno de los principales inconvenientes, que no está muy considerado, es la posible "brecha digital" de la población participante y también la general.

2º Calidad metodológica (De 0 a 8 puntos): alineamiento entre hipótesis y objetivos, formulados de forma clara y concisa; adecuación metodológica del diseño, variables y plan de análisis estadístico a los objetivos propuestos; tamaño muestral, justificación formal/informal; identificación de fuentes de sesgos y previsión para su control; propuesta de plan de gestión de datos (PGD).

Justifique la valoración otorgada

Existe una alineación correcta entre hipótesis y objetivos, estando ambos claramente detallados.
En general las variables de medida y la metodología están bien definidas aunque el plan de análisis estadístico no está muy desarrollado y puede que las herramientas estadísticas elegidas no sean las más pertinentes para cada uno de los parámetros a medir. Desde el punto de vista de los instrumentos, no se miden procesos cognitivos o de actividad, a pesar de que el programa se dirige a estos criterios de cambio. No quedan especificados todos los sesgos, p.ej., de la propia selección, los abandonos (una estimación, o un plan de contingencia). Se avala posibilidad de contar con muestra, pero no se especifica el control de su procedencia, más allá de los criterios de inclusión o exclusión. El plan de gestión de datos está bien detallado y es adecuado.

3º Viabilidad: (De 0 a 15 puntos): adecuación del equipo al proyecto, adecuación entre sus competencias y las actividades experimentales a desarrollar, experiencia previa; plan de trabajo, distribución de tareas y cronograma; identificación de riesgos y planificación de su control; infraestructuras disponibles, pertinencia y capacidad para su gestión; adecuación del presupuesto solicitado a los objetivos del proyecto; experiencia acreditada en gestión de proyectos, valoración específica en propuestas coordinadas o con presupuesto solicitado>300.000 euros.

Justifique la valoración otorgada

En general el equipo posee las competencias y la experiencia para llevar a cabo la mayoría de los objetivos propuestos. Quizá se eche en falta alguien con más experiencia científica y de gestión en la parte de metabolómica. Haría falta justificar mejor la razón de la externalización de los análisis estadísticos, que no se prevén especialmente complejos. En términos de viabilidad el trabajo ha de valorarse muy adecuadamente. Quizá la parte menos precisa es la de pérdida de muestra, el seguimiento y evaluación de las participantes. No queda claro cuánto es el apoyo o seguimiento telefónico, lo que puede convertirse en una variable extraña: no está especificado en su aplicación, sólo enunciado. A pesar del interés que tiene la propuesta, el presupuesto pedido es alto y las partidas están poco detalladas y justificadas.

Justifique brevemente la respuesta **respecto a valoración científica del proyecto**. Señale el grado de adecuación entre relevancia, aplicabilidad y capacidad de transferencia.

Interesante proyecto de continuación y de implementación de una herramienta online frente a la depresión. La valoración del proyecto es positiva en su relevancia, claramente aplicable, permitiendo, además, corroborar la utilidad de las intervenciones telemáticas (pero con presencia humana). Sin embargo según esta propuesta tiene elevado coste y mantenimiento de la herramienta, así como la falta de una justificación más detallada de los contenidos y de su creación, lo que podría dificultar su puesta en marcha y hacer más lento su potencial transferencia (que evidentemente tiene).

VALORACIÓN FINAL

Sólido proyecto de continuación que pretende implementar el uso de una herramienta online ya en funcionamiento frente a la depresión en mujeres con un grado moderado/leve de la misma (en mi opinión estaría bien comprobar si también puede funcionar en casos más graves de depresión). Como puntos fuertes cabe destacar la experiencia científica y de gestión del IP y de su equipo en este campo, que les hace ser referencias claras a nivel nacional y muy importantes a nivel internacional y la dimensión de género muy considerada. Como debilidades, falta un poco de explicación y experiencia sobre la parte metabolómica/hormonas sexuales, aunque se valora muy positivamente el haberse incluido como objetivos del proyecto. También se han identificado algunas carencias en el proyecto, más en su diseño y preparación, que en la idoneidad del equipo y la propia concepción del proyecto. En general destaca la falta de justificación y de detalle en algunas de las partidas presupuestarias, que no son bajas en algunos casos.

(Los datos personales que aparecen en este documento sólo serán utilizados para los fines previstos en la correspondiente Convocatoria o para su tratamiento automatizado con fines estadísticos con las garantías previstas en Reglamento (UE) 2016/679 del Parlamento Europeo y del Consejo de 27, de abril de 2016, relativo a la protección de las personas físicas en lo que respecta al tratamiento de datos personales y a la libre circulación de estos datos y por el que se deroga la Directiva 95/46/CE (Reglamento general de protección de datos) y en la Ley Orgánica 3/2018, de 5 de diciembre, de Protección de Datos Personales y garantía de los derechos digitales)

Fecha: jueves, 17 de agosto de 2023
