# Supplementary material for: A Women-Centered Internet-Based Cognitive Behavioral Therapy for Depressive Symptoms (the 3D Program): Protocol for a Randomized Controlled Trial
Source: JMIR Res Protoc. 2026 Aug 19;15:e99700. doi: 10.2196/99700 (PMC13489283; doi:10.2196/99700)
Supplement: Supplementary file 5 [file resprot-v15-e99700-s005.pdf]

DATOS GENERALES

**EXPEDIENTE**	PI23/00257
**INVESTIGADOR PRINCIPAL**	COLOM VICTORIANO, FRANCESC
**COINVESTIGADOR PRINCIPAL**	,
**TÍTULO**	Las mujeres luchan contra la depresión (WFD): una intervención basada en un programa online con interacción humana. Estudio de su eficacia e implicación metabolómica en la respuesta personalizada.
**CENTRO DE REALIZACIÓN**	INSTITUTO HOSPITAL DEL MAR DE INVESTIGACIONES MEDICAS (IMIM)
**CENTRO SOLICITANTE**	FUNDACION INSTITUTO HOSPITAL DEL MAR DE INVESTIGACIONES MEDICAS (FIMIM)
**COMISIÓN TÉCNICA DE EVALUACIÓN**	Enfermedades neurológicas y mentales

INFORME ESTRATÉGICO Y DE OPORTUNIDAD (máx 50 puntos)

A) Valoración del equipo de investigación (máx. 10 puntos).

Justifique brevemente la respuesta respecto a valoración estratégica del equipo de investigación.

Equipo formado por 7 investigadores (1 hombre y 6 mujeres) de una Institución acreditada como IIS. Si bien el CV del IP y de los colaboradores son muy destacados, no cuentan con IP o CO-IP con un contrato competitivo y/o menor de 45 años. Se concreta el aspecto de la participación ciudadana (email anexo) y la consecución de contratos Erasmus+, PFIS y Río Hortega confirman el aspecto de atracción de talento.

B) Valoración del proyecto (máx. 40 puntos).

1º Relevancia: (De 0 a 15 puntos): contribución esperada al avance de la ciencia en salud; estimación formal/informal del impacto (en salud, económico y social) por la implementación de sus resultados.

Justifique brevemente la respuesta.

En la redacción del proyecto justifica de forma solvente la relevancia del mismo, aportando cifras sobre la mayor frecuencia de la depresión en mujeres y sobre la baja disponibilidad de psicólogos por habitante 6/100.000 en el SNS, y apunta a ventajas del bajo coste de una intervención online para paliar este problema.

2º Aplicabilidad: (De 0 a 15 puntos): aplicabilidad directa o diferida, justificar la aplicabilidad directa, en caso de aplicabilidad diferida, considerar si se identifican retos regulatorios/organizativos a su implementación; vulnerabilidad del problema abordado; requiere modificaciones regulatorias u organizativas mayores.

Justifique brevemente la respuesta.

La aplicabilidad del proyecto es potencialmente directa: "en caso de obtener resultado positivo, el SNS contaría con un nuevo tratamiento coste-eficaz y fácil de implementar". Si bien esto es técnicamente plausible, requeriría de modificaciones regulatorias y organizativas.

3° Capacidad de transferencia del proyecto: (De 0 a 8 puntos): actuaciones previstas orientadas a la transferencia a la práctica clínica de los resultados, y su potencial incorporación en cartera de servicios; transferencia al sector productivo; limitaciones mayores para la transferencia efectiva.

Justifique brevemente la respuesta.

Se indica que, de resultar efectiva la intervención online WMD, su implementación es fácil.

4º Perspectiva de género: (De 0 a 2 puntos): ¿está incorporada en el diseño? ¿Es adecuado el planteamiento?

Justifique brevemente la respuesta.

El proyecto está dirigido solo a mujeres, atendiendo a su mayor incidencia de trastornos depresivos, siendo por tanto un planteamiento adecuado.

Justifique brevemente la respuesta respecto a la valoración estratégica del proyecto. Señale el grado de adecuación entre relevancia, aplicabilidad y capacidad de transferencia.

El proyecto es adecuado, relevante y de sencilla y coste-efectiva aplicabilidad si los resultados son positivos, si bien precisará de otros proyectos para garantizar su transferencia.

VALORACIÓN

Proyecto relevante y susceptible de ser financiado.

Fecha: martes, 25 de julio de 2023

Los datos personales que aparecen en este documento sólo serán utilizados para los fines previstos en la correspondiente Convocatoria o para su tratamiento automatizado con fines estadísticos con las garantías previstas en Reglamento (UE) 2016/679 del Parlamento Europeo y del Consejo de 27, de abril de 2016, relativo a la protección de las personas físicas en lo que respecta al tratamiento de datos personales y a la libre circulación de estos datos y por el que se deroga la Directiva 95/46/CE (Reglamento general de protección de datos) y en la Ley Orgánica 3/2018, de 5 de diciembre, de Protección de Datos Personales y garantía de los derechos digitales.
